# Characterizing the Phase-Structure and Rheological Response-Behavior of Multi-Walled Carbon Nanotubes Modified Asphalt-Binder

**DOI:** 10.3390/ma15134409

**Published:** 2022-06-22

**Authors:** Yongyi Li, Weijie Zhang, Chaoen Yin, Xiaorui Zhang

**Affiliations:** 1 College of Transportation Engineering, NJ Tech University, Nanjing 211816, China; hagongdadaolu@163.com; 2 School of Materials Science and Engineering, Southeast University, Nanjing 211189, China; wj_zhang9528@163.com

**Keywords:** asphalt-binder, phase-structure, rheological response-behavior, multi-walled carbon nanotubes, molecular simulation

## Abstract

In this study, the phase-structure and rheological response-behavior of multi-walled carbon nanotube (MWCNTs) modified asphalt-binder (MWCNTs-MA) were measured and quantified in the laboratory. The changes in the molecular dynamics due to MWCNTs modification were simulated and quantified based on the intermolecular interaction energy computations, electrostatic potential surface analyses and phase-structure modeling of the asphalt-binder matrix. The rheological properties such as the asphalt-binder viscosity and complex modulus, of both the base and modified asphalt-binders, were determined using the standard Brookfield viscometer (BV) and dynamic shear rheology (DSR) test devices, respectively. In comparison to the base asphalt-binder, the corresponding BV-DSR test results exhibited higher viscosity and complex modulus for the MWCNTs modified asphalt-binder, with reduced sensitivity and susceptibility to temperature variations. From the study results, it was observed that MWCNTs significantly improved the rheological properties and high-temperature performance of the asphalt-binder. Overall, the study has demonstrated that MWCNT modified asphalt-binder has great promising potential for application and usage as a road-pavement material, particularly with respect to mitigating the high temperature related distresses such as rutting.

## 1. Introduction

Among others, asphalt pavements have the advantages of low noise, good skidding and wearing resistance, convenience of maintenance and repair, flat surface with no joints, slight traffic vibration and rapid construction for the safe and comfortable driving contributed by the asphalt-binder, a viscoelasticity material [1,2,3]. Asphalt concrete is thus widely used in pavement construction, accounting for over 90% of the total highway mileage [4]. Nonetheless, asphalt pavements are subjected to numerous traffic loads and complex environmental changes during their service life, which inevitably results in gradual pavement deterioration, including enduring distresses such as rutting, cracking and moisture damage. To improve the properties and optimize the performance of the base asphalt-binder, modified asphalt-binders are increasingly being used to enhance the longevity and durability of asphalt pavements.

Polymer is one of the commonly used asphalt-binder modifiers, with the modification achieved through the formation of stable colloids caused by component changes such as saturates, aromatics, resins, asphaltenes (SARA), etc. [5,6]. These polymer modifiers include styrene-butadiene-styrene (SBS), Styrene-Butadiene Rubber (SBR), ethylene-vinyl acetate (EVA), etc. [5,6]. Ordinary polymer modified asphalt-binder is generally prone to thermal and ultraviolet (UV) aging, as well as segregation in the process of production, storage and operation, due to the significant difference in the density, microstructure and molecular polarity between the polymer and the base asphalt-binder that greatly limits its engineering applicability [7,8].

Based on Stoke’s Law, SBS has an inclination to float in the base asphalt-binder, which may eventually contribute to the formation of a thick congealed layer of SBS-rich phase at the surface of the SBS polymer asphalt-binder [9]. Therefore, some new asphalt-binder modification methods, in addition to polymer modification, are continuously being explored to enhance the rheological, mechanical, physical and other macro properties of the asphalt-binder.

Carbon nanotubes (CNTs) are widely applied in many fields due to their extremely high tensile strength and ductility, as well as remarkable electrical and thermal properties [10,11,12]. The addition of CNTs reduces the rate of decomposition and volatilization of saturates and aromatics, which ultimately contributes to the better thermal stability of the asphalt-binder [13]. Furthermore, CNTs also have the characteristic property of having a large specific surface area that not only aids to form complex network microstructures, but also helps to enhance the adhesive bonding at the interface between the base asphalt-binder and aggregates [14,15]. Compared with single-walled carbon nanotubes (SWCNTs), multi-walled carbon nanotubes (MWCNTs) have stronger surface activity that can potentially contribute to enhancing the rheological, physical and mechanical properties of the base asphalt-binder. In this study, the asphalt-binders were modified with MWCNTs to enhance their rheological and physical properties.

Thus far, base asphalt-binders with different modification additives have been vastly investigated in the field of rheology [16,17,18,19,20,21]. Airey et al. [16,17] investigated the rheological response-behavior of SBS polymer-modified bitumen (PMB) using the Dynamic Shear Rheometer (DSR) test device. From their study [16,17], it was found that the modification degree of SBS is related to the asphalt-binder or bitumen source, asphalt-binder polymer compatibility and polymer concentration, and the findings indicated a modification enhancement of the high-temperature resistivity properties through an increase in the viscosity, stiffness and elastic response-behavior of the PMB.

Xia et al. [18] studied the morphological evolution and viscoelastic change of SBS polymer-modified asphalt-binders with different SBS mass fractions. Shan et al. [19] studied the non-linearity of asphalt-binders using Fourier Transform (FT)-rheology and strain decomposition methods. The study revealed that SBR can potentially increase the performance of modified asphalt-binders at high temperatures due to the large amount of carbon blocks [20]. However, the SBR was found not to be as effective as SBS in enhancing the high and low temperature properties of the base asphalt-binder [22]. González et al. [21] analyzed the rheology of a bitumen modified with high- and low-density polyethylene and their respective blends with ethylene/propylene/ethylidene norbornene. The rheological results [23] indicated a significant increase in the elastic modulus and complex viscosity of the modified bitumen.

In conclusion, polymer phase distribution characteristics affect the microstructure, which means the interface between the polymer and modified asphalt is still weak. On the other side, the correlation between the microstructure and rheological properties of polymers is not clear. Therefore, it is necessary to study on interface strengthening mechanism of MWCNTs-MA based on molecular dynamics to obtain the possibility of interface enhancement of MWCNTs-MA. In addition, the MWCNTs dosage have the influence on performance of asphalt binder, which needs to analysis the rheological properties of MWCNTs-MA at different temperatures. In this laboratory study, the phase-structure and rheological response-behavior of multi-walled carbon nanotube (MWCNTs)-modified asphalt-binder (MWCNTs-MA) were measured and quantified. The changes in the molecular dynamics due to MWCNTs modification were simulated and quantified based on the intermolecular interaction energy computations, electrostatic potential surface analyses and phase-structure modeling of the asphalt-binder matrix. The rheological properties such as the asphalt-binder viscosity and complex modulus, for both the base and modified asphalt-binder, were determined using the standard Brookfield Rotational Viscometer (RV) and DSR test devices, respectively.

In the subsequent sections of the paper, the experimental plan, materials used, molecular simulations and laboratory testing are discussed, followed by a presentation of the results and an analysis thereof. The paper then concludes with a summary of key findings and recommendations.

## 2. Experimental Plan and Materials Used

As aforementioned, the primary objective of this study was to modify a typical Chinese asphalt-binder with MWCNTs, and thereafter quantify its resultant phase-structure and rheological response-behavior. To address these objectives, the experimental plan incorporated the following five key tasks: (a) sourcing and acquiring the materials, namely the asphalt-binder and Carbon Nanotubes; (b) preparing the asphalt-binder samples; (c) molecular simulations and modeling; (d) laboratory experimentation and testing; and (e) data analysis and synthesis. The materials used in the study, along with sample preparation, are discussed in this section of the paper. The rest of the tasks are presented and discussed in the subsequent texts.

### 2.1. Base Asphalt-Binder (A-70#)

The base asphalt-binder used in this study was a typical A-70# Petroleum asphalt-binder commonly used and produced in China. The physical and rheological properties (technical indices) of the base asphalt-binder that were tested based on the Chinese Test Specification JTG E20-2011 [24] are listed in Table 1.

As shown in Table 1, the base asphalt-binder satisfactorily met the requirements in accordance with the Chinese technical specifications for construction of highway asphalt pavements (JTG F40-2004) [25]. Note that in this paper, the terms asphalt, binder, asphalt-binder, bitumen and bituminous binder have been used interchangeably.

### 2.2. Carbon Nanotubes (MWCNTs)

The MWCNTs used in this study were sourced from the Chinese Academy of Sciences in China. The key characteristic properties and technical indices of MWCNTs are listed in Table 2.

### 2.3. Asphalt-Binder Modification and Sample Preparation

Dispersion of MWCNTs in the asphalt-binder matrix is a major challenge due to its high surface area and aspect ratio. In this study, the high-speed homogeneous shearing method [26] was used for dispersion. Initially, a 300 g base asphalt-binder was heated to flow at 155 ± 5 °C and divided into vessels of the same size. Thereafter, the asphalt-binder was mixed alone without CNT addition at a shear speed of 5000 revolutions per minute (rpm) for 5 min, in order to ensure uniform temperature distribution.

For the modification process, the proportions of MWCNTs were arbitrarily selected as 0.5%, 1.0%, 1.5%, 2.0% and 2.5%, respectively, by the weight of the base asphalt-binder. These proportions were subsequently slowly added into the control asphalt-binder matrix to minimize the agglomeration of the added MWCNTs. Mixing of the asphalt-binder with each MWCNTs dosage was conducted at a shear speed of 5000 rpm for 30 min, in a 155 ± 5 °C oil-bath, to complete the mixing process.

After shear mixing, the modified asphalt-binder blend with each respective MWCNTs percentage/dosage in the oil-bath for 30 min to stabilize it prior to laboratory testing and molecular simulations. For each MWCNTs dosage, a minimum of three sample replicates were prepared per test condition and per test type.

## 3. Molecular Simulations and Laboratory Testing

The molecular simulations and modeling of the MWCNTs modified asphalt-binder matrix are described in this section of the paper. The laboratory tests for characterizing the rheological properties of the MWCNTs modified asphalt-binder are also discussed, and also included are the RV (for viscosity measurements) and temperature frequency-sweep tests using the DSR test device for modulus measurements.

### 3.1. Modeling and Molecular Simulations

Molecular simulation and phase-structure modeling of the MWCNTs modified asphalt-binder are discussed below. The phase-structure modeling included simulation of the key constituent components of the both the base and MWCNTs-modified asphalt-binder, as well as molecular dynamics and intermolecular interactions.

#### 3.1.1. Phase-Structure Modeling and Molecular Simulations

**Figure 1:**
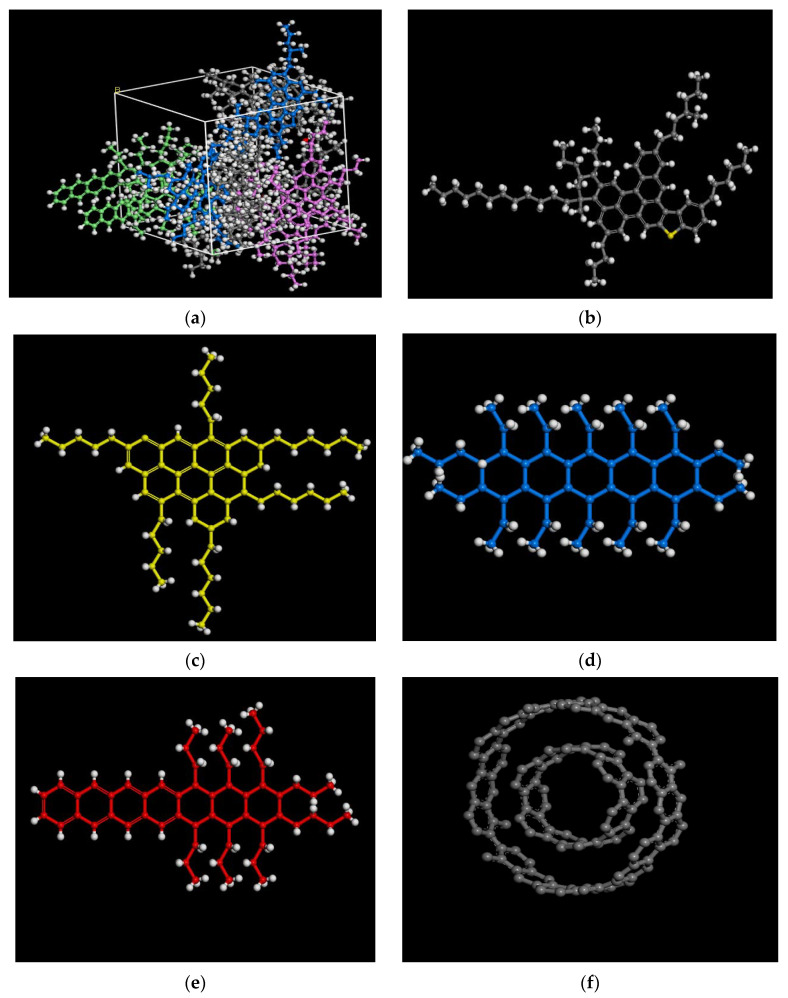
Molecular models of MWCNTs-MA: (**a**) asphalt-binder; (**b**) asphaltenes; (**c**) resins; (**d**) saturates; (**e**) aromatics; and (**f**) MWCNTs.

The Materials Studio (MS), an Accelrys commercial software, was employed to simulate the phase-structure and rheological response-behavior of the MWCNTs asphalt-binder, denoted as MWCNTs-MA. As shown in Figure 1a, the base asphalt-binder typically comprises different constituent compounds, mainly the saturates, aromatics, resins and asphaltenes, or SARA. From the literature reviewed by these authors, the most widely used analysis method for simulating these constituent compounds (namely SARA) is the four-component analysis method based on the ASTM D4124-09 standard [27]. This was the method adopted in this study, and the four components (i.e., SARA) were used as the inputs for modeling the phase-structure of the base asphalt-binder [28].

As shown in Figure 1b, the asphaltenes molecules were modeled and represented using the chemical formular C_76_H_98_S. The resins, on the other hand, were represented using the C_60_H_89_N molecular model, and are as shown in Figure 1c. These resins can be used as a solvent for the asphaltenes.

The saturates, which have the highest content of long-chain alkanes in the asphalt-binder, were represented using the C_51_H_94_ molecular model shown in Figure 1d. As shown in Figure 1e, C_56_H_82_ was used as a representative molecule for the aromatics. In this study, the number of walls and wall separation of MWCNTs were 2 and 3.347, respectively; see Figure 1f.

#### 3.1.2. Phase-Structure Dynamics and Intermolecular Interactions

The macro phenomena of an asphalt-binder are not only a function of its micro properties, but its ultimate performance is also dependent on these micro properties when in normal state [29]. The intermolecular interaction of energy, *E_inter_*, within the asphalt-binder can be determined using the Cohesive Energy Density (CED) as expressed in Equation (1) below [30]

(1) $$E_{A-B}=E_{total}-E_{A-A}-E_{B-B}$$

where *E_total_* is the total cohesive energy density, *E_A-A_* is the cohesive energy density of the A molecules and *E_B-B_* is the cohesive energy density of the *B* molecules. 

The solubility parameter (*δ*) indicates the degree of compatibility among substances/compounds, and it also reflects the degree of stability of the blended system [31]. The *δ* parameter can be computed using Equation (2) below [30,31]:

(2) $$\delta =\sqrt{CED}=\sqrt{\frac{\Delta E}{V}}$$

In Equation (2), *δ* is the solubility parameter, *CED* is cohesive energy density, Δ*E* is cohesive energy and *V* is the volume of the blended system. The electrostatic potential surface was calculated using the first principle of the generalized gradient approximation and density-functional exchange-energy approximation with correct asymptotic behavior [32]. The phase-structure change was evaluated based on the surface area concepts and the occupied volume. The total volume is generally a constant, so the larger the occupied volume of the molecules, the more the molecules will aggregate together.

### 3.2. Brookfield Rotational Viscosity (RV) Testing

The viscosity of the asphalt-binder at high temperatures (above 135 °C) is considered as an important characteristic property, as it expresses the ability to pump fluid asphalt-binder into a hot-mix asphalt (HMA) manufacturing plant [33]. The viscosity property also aids the asphalt-binder to completely coat the aggregate during mixing, and allows the easy compaction of the asphalt-mixture.

In this study, the Brookfield rotational viscometer (RV) test was used to measure the viscosity of the unmodified and MWCNTs-modified asphalt-binder samples at three temperatures (namely 135, 150 and 165 °C, respectively, in accordance with the ASTM D4402 test specification) [34]. Taking the segregation of modified asphalt-binder into consideration, a minimum of three sample replicates were tested per asphalt-binder per test RV condition.

### 3.3. Temperature Frequency-Sweep Tests Using the DSR Device

Temperature frequency-sweep tests were conducted to measure the rheological properties of the modified asphalt-binder, namely the complex modulus. The tests were performed in accordance with the ASTM D7175 (ASTM 2015c) test standard using the DSR device (SYD-0628, HT Company, New Delhi, India) [35]. The test temperature ranged from 46 °C to 88 °C at an incremental rate of 6 °C and a constant frequency of 10 rad/s. The DSR parallel plate geometry comprised a 25-mm diameter and 1-mm gap for holding the sample.

According to the Strategic Highway Research Program and Superpave specifications [36,37], the values of G*/Sin *δ* for original unaged asphalt-binder and short-term-aged asphalt-binder are limited to 1.0 kPa and 2.2 kPa, respectively [38]. Like the RV test, a minimum of three sample replicates were tested per asphalt-binder per DSR test condition.

## 4. Results, Analyses, Synthesis and Discussions

The molecular modeling and laboratory test results are presented, analyzed and synthesized in this section. As discussed below, these results include the intermolecular interaction energy computations/quantification, electrostatic potential surface analyses, matrix phase-structure modeling and the rheological properties, namely the viscosity and complex modulus of asphalt-binder.

### 4.1. Intermolecular Interaction Energy Quantification

The intermolecular interaction energy between MWCNTs and the four constituent components, namely SARA, are evaluated and discussed in the subsequent text. Generally, the uneven distribution of the asphalt-binder in the blended mix was assumed to occur based on the hypothesis that the intermolecular interaction energy of the different molecules are different, and so is the dynamic movement. The corresponding CED, intermolecular interaction energy and solubility results of MWCNTs-MA are shown in Table 3.

As shown in Table 3, with respect to base asphalt, the CED absolute value for the aromatics-aromatics pair was the largest, seconded by the saturates-saturates pair, while the CED absolute value of the asphaltenes-asphaltenes pair was in third position. The smallest and least CED absolute value was registered for the resins-resins pair. These results indicate that the intermolecular interaction force and affinity of the aromatics-aromatics pair was larger than the other pairs, while that of the resins-resins pair was the smallest.

The literature has reported a connection between solubility (*δ*) and CED for similar molecules that could be soluble within each other, i.e., inter-soluble. That is the closer the solubility parameter in magnitude, the more compatible and soluble the molecules are with each other. With this in mind, the *δ* results in Table 3 indicate that asphaltenes is easily soluble into resins rather than in aromatics or saturates. On the other hand, the closeness of the *δ* values for the saturates and aromatics, i.e., 14.12–14.90 (J/cm^3^)^1/2^, suggests that the aromatics are easily soluble into the saturates and vice versa.

With respect to the MWCNTs, the *E_inter_* absolute value of the MWCNTs-saturates pair was the largest, followed by the *E_inter_* absolute value of the MWCNTs-resins pair whilst that of the MWCNTs-asphaltenes pair was the smallest. The intermolecular interaction energy in MWCNTs-MA seems to follow a similar trend with respect to the solubility parameter—that is, the lower the *δ* value, the more soluble the MWCNTs is in that constituent compared. From Table 3, it can thus be inferred that MWCNTs is most easily dissolvable into the aromatics, consecutively followed by the saturates, asphaltenes and resins. Overall, these results suggest that MWCNTs may not be easily soluble into the asphalt-binder, as resins constitute the main solvent in the asphalt-binder system.

### 4.2. Electrostatic Potential Surface Analysis and Characterization

**Figure 2:**
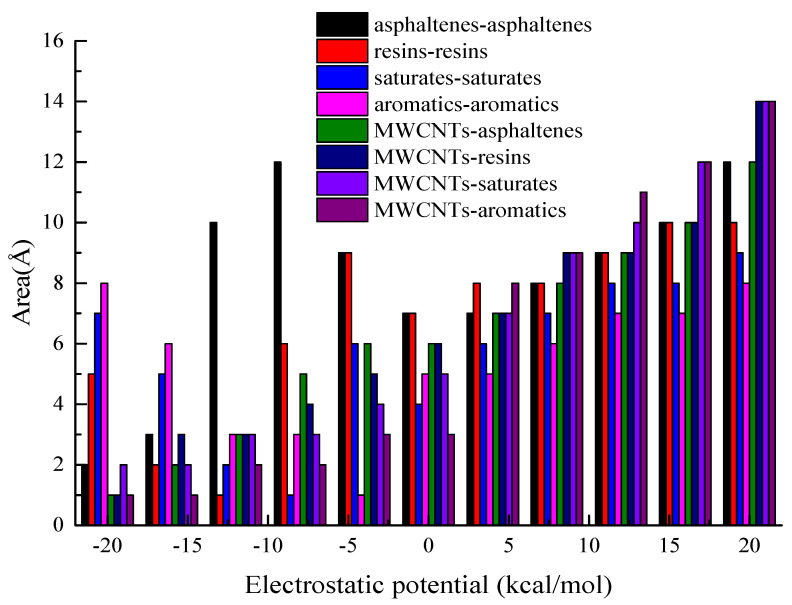
Charge distribution of MWCNTs-MA.

The electric clouds of the aromatics, resins and asphaltenes are distributed on the aromatic rings, and there exists some huge π-π interactions. The electrostatic potential surface of MWCNTs and the four constituent components, namely SARA, are shown in Figure 2.

During experimentation and molecular modeling, the electric clouds of the MWCNTs appeared on the interlayer and tubes. Furthermore, the electric clouds of MWCNTs showed a positive charge, with the inner layer exhibiting a negative charge. This indicated that the positive charge is easy to move to the outer layer of the MWCNTs, which can lead to an uneven distribution of the charge. The charge distribution of the side chains for the saturates, aromatics and asphaltenes are even, with the intermolecular interaction force being predominantly the Van der Waals’ (vdW) forces [39,40]. Therefore, the MWCNTs could potentially enhance the electric clouds density of the aromatics and resins, while simultaneously decreasing the electric clouds density of the resins and asphaltenes.

The charge distribution of the MWCNTs-aromatics and MWCNTs-saturates mainly comprise the π-π interaction force. Overall, the results indicated that MWCNTs can improve the conductivity of the asphalt-binder and the corresponding asphalt-mixtures, especially aromatics and saturates. Furthermore, it was also found that MWCNTs could potentially improve the polarity (π-π interaction force) of asphalt-binder, particularly the aromatics and saturates. On the other hand, it was observed that MWCNTs did not have a significant effect on the electric cloud density and charge distribution of the resins—that is, the effect was marginal.

### 4.3. Phase-Structure Modeling of the Asphalt-Binder Matrix

**Figure 3:**
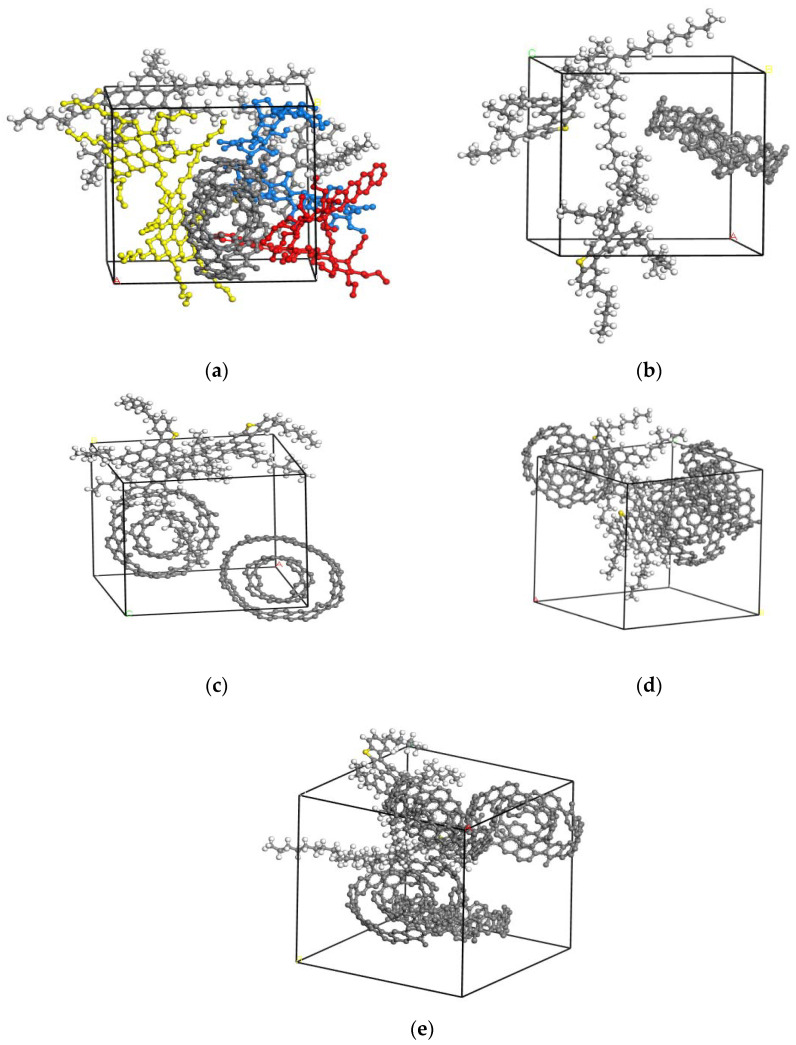
The aggregate or collective-solid states of the MWCNTs-MA matrix: (**a**) 1.0% MWCNTs; (**b**) 1.0% MWCNTs deleted resins, saturates and aromatics; (**c**) 1.5% MWCNTs deleted resins, saturates and aromatics; (**d**) 2.0% MWCNTs deleted resins, saturates and aromatics; and (**e**) 2.5% MWCNTs deleted resins, saturates and aromatics.

In the molecular modeling of the asphalt-binder matrix, the phase-structure change can be evaluated using surface area, the occupied volume and aggregate (collective) states. To better understand the aggregates or collective-solid states of the MWCNTs-MA matrix, the molecule numbers of each of the four constituent components (namely SARA) were two, whilst the molecule numbers of MWCNTs ranged from 1.0 to 4.0. The aggregate (or collective-solid) states of the MWCNTs-MA matrix with different MWCNTs content are shown in Figure 3.

As shown in Figure 3, the aggregate or collective-solid states of MWCNTs and asphaltenes was the main reason for the uneven molecular distribution. As evident in Figure 3b through to Figure 3e, the aromatics, saturates and resins molecules were hidden/removed to allow for the discrete modeling of the asphaltenes and MWCNTs aggregation. In general, the aggregation of the asphaltenes increased with the corresponding distance between the asphaltenes and MWCNTs becoming smaller as the content of MWCNTs increased. This indicates that the stability of the system becomes relatively poor and easy to precipitate and separate into phases. There is an interesting phenomenon that the MWCNTs-MWCNTs and MWCNTs-asphaltenes tend to coalesce towards a parallel arrangement with an increase in the MWCNTs content. True to this theory, it was observed in this study that the MWCNTs-MA matrix amalgamated towards a parallel arrangement with an increase the MWCNTs content, ultimately substantiating that MWCNTs could substantially improve the aggregation arrangement of the molecules in the MWCNTs-MA matrix.

The surface area and occupied volume of the MWCNTs-MA matrix were computed based on molecular dynamic simulations, and are shown in Table 4. As listed in the table, the surface area and free volume increased with an increase in the MWCNTs content, indicating that MWCNTs could potentially improve the vdW interaction forces within the MWCNTs-MA matrix, and thus enhance the molecular movement. Theoretically, this means that the higher the MWCNTs content is, the easier it is for the MWCNTs molecular movement.

Like surface area and free volume, the results in Table 4 also show that the occupied volume exhibited an increasing response trend with an increase in the MWCNTs content. This response trend suggests that MWCNTs will potentially improve the vdW interaction forces within the MWCNTs-MA matrix. That is, the vdW interaction forces will also proportionally increase as a function of the MWCNTs content. The most important aspect of these results is that MWCNTs could significantly affect the phase-structure of MWCNTs-MA matrix, particularly the surface area of the vdW and the occupied volume of the vdW.

**Figure 4:**
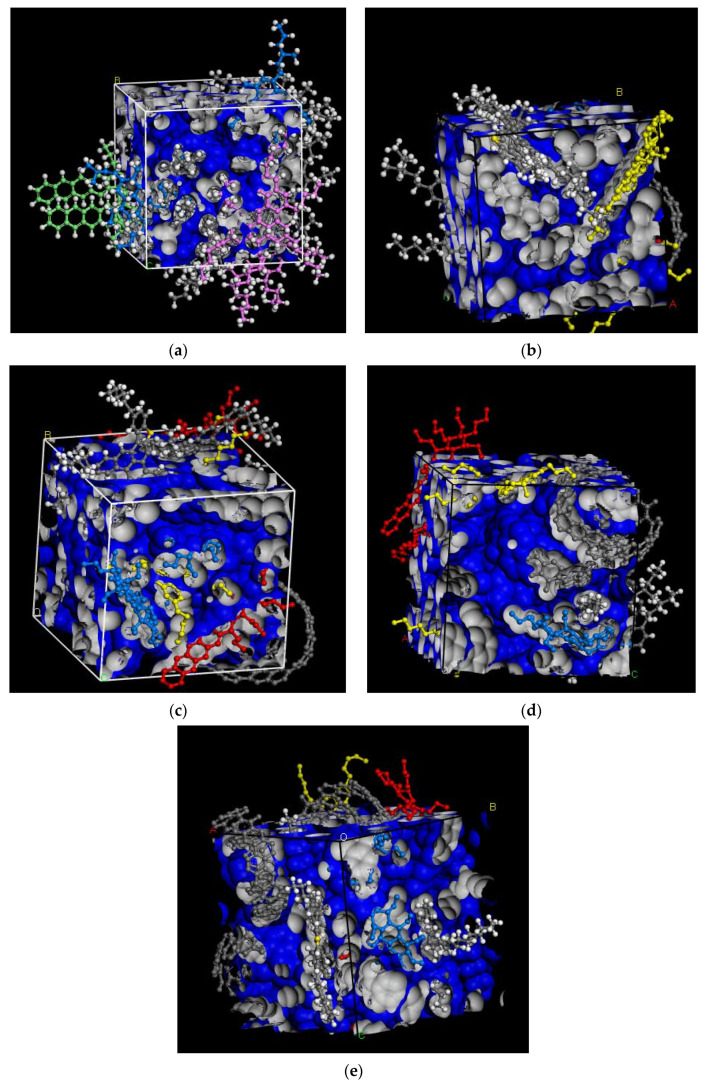
Free volume modeling of the MWCNTs-MA matrix: (**a**) Asphalt-binder; (**b**) 1.0% MWCNTs-MA; (**c**) 1.5% MWCNTs-MA; (**d**) 2.0% MWCNTs-MA; and (**e**) 2.5% MWCNTs-MA.

As shown in Figure 4, it can clearly be visually seen that the free volume increased with an increase in the MWCNTs content. The figure furthers suggests that the molecular movement of 1.5% MWCNTs-MA was easier than that of the 1.0% MWCNTs-MA matrix.

Other than MWCNTs, the distribution of all the other molecules changed significantly with an increase in the MWCNTs content. Overall, the results in Figure 4 indicate that MWCNTs would significantly affect the inner structure of the MWCNTs-MA matrix, with the effect being more prominent as the MWCNTs content increases. Based on the hypothesis that total volume is constant, the free volume will correspondingly increase as the MWCNTs content is increased. These partially prove that the MWCNTs improved the inner structure rearrangement of the MWCNTs-MA matrix.

### 4.4. Viscosity-Temperature Characterization of MWCNTs-MA

**Figure 5:**
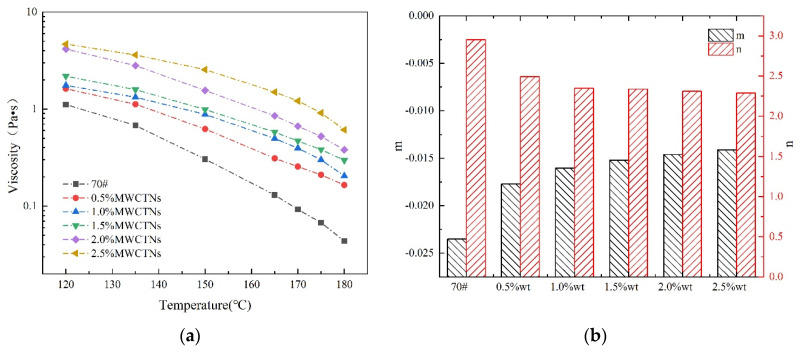
(**a**) Viscosity-temperature response curves of MWCNTs-MA; (**b**) Curve slope for VTS.

To verify the molecular modeling and simulation results, the viscosity-temperature response curves of the MWCNTs-MA were measured and characterized using the Brookfield viscometer (RV) test. As shown in Figure 5, the viscosity changed significantly with the change in temperature and MWCNTs content, i.e., it decreased as a function of temperature and vice versa for MWCNTs content.

More theoretically expected, Figure 5 shows that the MWCNTs-modified asphalt binder exhibited higher viscosity than the base asphalt-binder at all temperatures. For MWCNTs-MA, the optimal mixing and paving temperatures are 125 °C and 165 °C, respectively. Due to the very high specific surface area and surface energy of MWCNTs compared with asphalt binder as shown in Table 4, polymer SBS can adsorb MWCNTs to form the interface phase between SBS and asphalt binder, which leads to more stable synergistic system with stronger deformation resistance. Previous studies have found that the viscosity could reflect the inner structure of the asphalt-binder, and the higher the viscosity in magnitude, the harder the inner structure will become. The results in Figure 5 suggest that MWCNTs and high temperature would increase the inner structure of modified asphalt-binder, and significantly affect its phase-structure or molecular distribution. These test results are like the simulation results in Figure 4. Overall, these results indicate that the phase structure and rheological response-behavior of MWCNTs modified asphalt-binder can be investigated using molecular simulations. Thus, molecular modeling and simulation constitute a promising method to simulate the phase-structure and rheological response-behavior of MWCNTs-MA.

### 4.5. Rheological Response-Behavior of MWCNTs-MA

**Figure 6:**
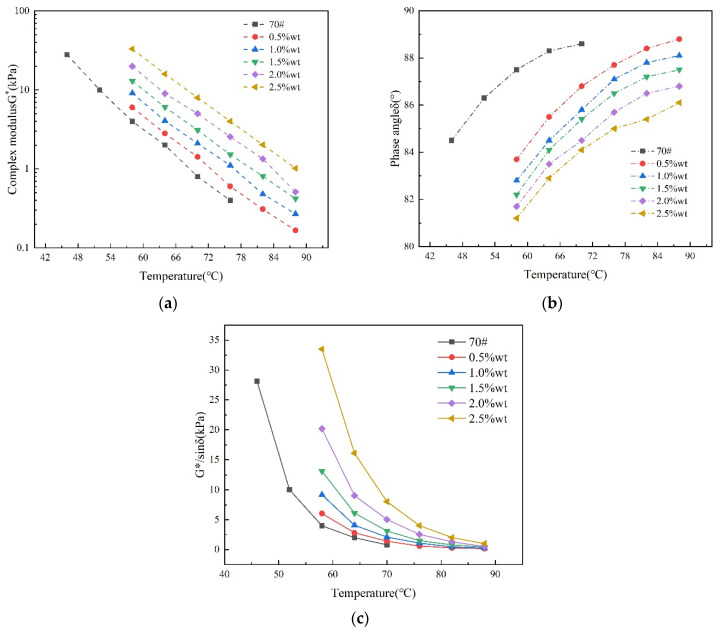
Temperature frequency-sweep response curves of MWCNTs-MA: (**a**) Complex modulus; (**b**) Phase angle; (**c**) Rutting factors.

The temperature frequency-sweep response curves of MWCNTs-MA are shown in Figure 6 (1.59 Hz). With the increase of temperature, the complex modulus of MWCNTs-MA decreased, while the complex modulus of MWCNTs-MA increased with an increase in the MWCNTs content. The test results showed that the MWCNTs content could improve the high-temperature anti-rutting properties of MWCNTs-MA, and decreased the flow properties of MWCNTs-MA.

For this study, the rutting temperature of MWCNTs-MA was computed at a rutting factor of 1.0 kPa. With this consideration, the rutting temperature for 1.0% MWCNTs-MA, 1.5% MWCNTs-MA, 2.0% MWCNTs-MA and 2.5% MWCNTs-MA were determined to be 45, 60, 65, 70 and 72 °C, respectively, from Figure 6. These results indicate that MWCNTs can potentially improve the rutting temperature resistance properties of MWCNTs-MA, and that this MWCNTs modification can enhance the high-temperature anti-rutting properties of the asphalt-binder. Overall, these findings substantiate that MWCNTs can potentially improve the rheological response-behavior of MWCNTs-MA, and thus be able to verify the simulated results.

## 5. Conclusions and Recommendations

This study investigated the phase-structure and rheological response-behavior of multi-walled carbon nanotubes-modified asphalt-binder using molecular simulations and laboratory experimentations. A typical Chinese A-70# Petroleum asphalt-binder was used as the base asphalt-binder. MWCNTs were used as the asphalt-binder modifier. From the study results and findings, the following conclusions and recommendations were drawn:

MWCNTs is mostly soluble in aromatics than in saturates, asphaltenes and resins. Additionally, MWCNTs is not highly soluble in asphalt-binder due to the high presence of resins that serve as the solvent in asphalt-binder system.

MWCNTs can potentially improve the conductivity of the asphalt-binder and asphalt mixtures, especially aromatics and saturates. Additionally, MWCNTs can also improve the polarity (π-π interaction force) of the asphalt-binder, especially aromatics and saturates. By contrast, MWCNTs had marginal effect on the electric cloud density and charge distribution of the resins.

MWCNTs-MA coalesced towards a parallel structure arrangement with an increase in the MWCNTs content, indicating that MWCNTs has the potential to improve the aggregation arrangement of the MWCNTs-MA matrix. The study further indicated that MWCNTs had a significant effect on the molecular dynamics and phase-structure of the MWCNTs-MA matrix, particularly the surface area and occupied volume of vdW.

MWCNTs and high temperature increased the inner structure change of MWCNTs-MA, with a significant effect on the phase-structure or molecular distribution within the MWCNTs-MA matrix. From comparisons with laboratory experimentations, it was concluded that the phase-structure and rheological response-behavior of MWCNTs-MA can be characterized and quantified using molecular simulations. Thus, molecular modeling constitutes a potentially promising methodology for simulating the phase-structure and rheological response-behavior of MWCNTs-MA.

In comparison to the base asphalt-binder and as theoretically expected, the laboratory test results yielded higher viscosity and complex modulus for the MWCNTs modified asphalt-binder, with reduced sensitivity and susceptibility to temperature variations. That is, the MWCNTs significantly improved the rheological properties and high-temperature performance of the asphalt-binder.

Overall, the study has demonstrated that MWCNT-modified asphalt-binder has great promising potential for application and usage as a road-pavement material, particularly with respect to mitigating the high temperature related distresses such as rutting. However, the results and findings pertain to the materials, test methods and simulation models used in the study; therefore, the overall conclusions may not be exhaustive nor exclusive. It is thus recommended that future studies should incorporate a wide array of materials (i.e., different asphalt-binders and CNTs modifiers), simulation models and laboratory test methods include moisture and crack susceptibility evaluations, along with field validation, to further supplement the findings reported herein. Nonetheless, this study beneficially contributes to the literature through provision of a reference datum for using MWCNTs as a modifier, including molecular simulation of the resultant MWCNTs-MA matrix.

## Figures and Tables

**Table 1:** Technical indexes of A-70# Petroleum asphalt-binder.

Technical Indices		Unit	Specification (JTG F40-2004)	Test Results
**Penetration (25 °C, 100 g, 5 s)**		0.1 mm	60~80	61
Penetration index, PI		—	−1.5~+1.0	−1.45
Softening point, T_R&B_		°C	≥46	46.2
Ductility (15 °C, 5 cm/min)		cm	≥100	>100
Ductility (10 °C, 5 cm/min)		cm	≥15	22.51
Density @15 ℃		g/cm^3^	/	1.043
Wax content		%	<2.20	2.18
Dynamic viscosity @60 ℃		Pa∙s	≥180	248
Kinematic viscosity @135 ℃		Pa.s	/	0.418
After RTFOT (163 ℃, 85 min)	Mass change	%	−0.8~+0.8	0.05
	Penetration ratio @25 °C	%	≥61	73.85
	Ductility (10 °C, 5 cm/min)	cm	≥6	9

Legend: RTFOT = Rolling thin film oven test; T = Temperature; R&B = Ring and Ball.

**Table 2:** Key characteristic properties and technical indices of MWCNTs.

Item	Values
Average length (μm)	20
Bulk density (g/cm^3^)	0.020~0.100
Surface area (m^2^/g)	250~300
Average diameter (nm)	6.0
Young’s modulus (TPa)	≥1.0
Metal particle content (%)	<1.0
Amorphous carbon content (%)	<1.0
Production method	Chemical vapor deposition (CVD)

**Table 3:** Intermolecular interaction energy results of MWCNTs-MA.

Inter-Molecular Pair	CED (J/m^3^)	*E_inter_* (kJ/mol)	*δ* (J/cm^3^)^1/2^
Asphaltenes-asphaltenes	2.96 × 10^8^	−756.25	17.10
Resins-resins	2.63 × 10^8^	−656.12	16.20
Saturates-saturates	1.99 × 10^8^	−1485.65	14.12
Aromatics-aromatics	2.22 × 10^8^	−3347.76	14.90
MWCNTs-asphaltenes	2.40 × 10^8^	−1841.25	15.50
MWCNTs-resins	2.29 × 10^8^	−2352.62	15.12
MWCNTs-saturates	1.93 × 10^8^	−3425.25	13.89
MWCNTs-aromatics	1.87 × 10^8^	−2125.15	13.67

**Table 4:** Effects of MWCNTs content on molecular aggregation.

Sample	Surface Area (Å²)	Occupied Volume (Å³)	Free Volume (Å³)
Asphalt-binder	5103.31	8127.41	1887.89
1.0% MWCNTs-MA	7540.74	7424.08	5342.92
1.5% MWCNTs-MA	8791.06	8806.64	6712.74
2.0% MWCNTs-MA	10,235.24	10,190.48	8081.27
2.5% MWCNTs-MA	11,454.22	11,562.91	9461.21

## Data Availability

The data presented in this study are available on request from the corresponding author.

## Abbreviations

MWCNTs	Multi-walled Carbon Nanotubes
MA	modified asphalt-binder
BV	Brookfield viscometer
DSR	dynamic shear rheology
SARA	saturates, aromatics, resins, asphaltenes
SBS	styrene-butadiene-styrene
SBR	Styrene-Butadiene Rubber
EVA	ethylene-vinyl acetate
UV	ultraviolet
PMB	polymer modified bitumen
FT	Fourier Transform
RV	Rotational Viscometer
RTFOT	Rolling thin film oven test
CVD	Chemical vapor deposition
MS	Materials Studio
CED	Cohesive Energy Density
HMA	hot-mix asphalt
vdW	Van der Waals

## References

[1] Kane M., Edmondson V. (2020). Long-term skid resistance of asphalt surfacings and aggregates’ mineralogical composition: Generalisation to pavements made of different aggregate types. Wear.

[2] Rahman T., Dawson A., Thom N. (2020). Warm mix asphalt (WMA) for rapid construction in airfield pavement. Constr. Build. Mater..

[3] Hernandez-Fernandez N., Underwood B.S., Ossa-Lopez A. (2020). Simulation of the asphalt concrete stiffness degradation using simplified viscoelastic continuum damage model. Int. J. Fatigue.

[4] Cui P., Wu S., Xiao Y., Yang C., Wang F. (2019). Enhancement mechanism of skid resistance in preventive maintenance of asphalt pavement by steel slag based on micro-surfacing. Constr. Build. Mater..

[5] Sengoz B., Isikyakar G. (2008). Analysis of styrene-butadiene-styrene polymer modified bitumen using fluorescent microscopy and conventional test methods. J. Hazard. Mater..

[6] Hu K., Han S., Liu Z., Niu D. (2019). Determination of morphology characteristics of polymer-modified asphalt by a quantification parameters approach. Road Mater. Pavement Des..

[7] Xiang L., Cheng J., Que G. (2009). Microstructure and performance of crumb rubber modified asphalt. Constr. Build. Mater..

[8] Polacco G., Filippi S., Merusi F., Stastna G. (2015). A review of the fundamentals of polymer-modified asphalts: Asphalt/polymer interactions and principles of compatibility. Adv. Colloid Interface Sci..

[9] Golestani B., Nam B.H., Nejad F.M., Fallah S. (2015). Nanoclay application to asphalt concrete: Characterization of polymer and linear nanocomposite-modified asphalt binder and mixture. Constr. Build. Mater..

[10] Hayashi T., Kim Y.A., Natsuki T., Endo M. (2007). Mechanical properties of carbon nanomaterials. Chemphyschem.

[11] Chen J., Yan L. (2018). Effect of carbon nanotube aspect ratio on the thermal and electrical properties of epoxy nanocomposites. Fullerenes Nanotub. Carbon Nanostruct..

[12] Sarangdevot K., Sonigara B.S. (2015). The wondrous world of carbon nanotubes: Structure, synthesis, properties and applications. J. Chem. Pharm. Res.

[13] Shu B., Wu S., Pang L., Javilla B. (2017). The utilization of multiple-walled carbon nanotubes in polymer modified bitumen. Materials.

[14] Khalid A., Al-Juhani A.A., Al-Hamouz O.C., Laoui T., Khan Z., Atieh M.A. (2015). Preparation and properties of nanocomposite polysulfone/multi-walled carbon nanotubes membranes for desalination. Desalination.

[15] Tarefder R.A., Zaman A. (2017). Carbon nanotube modified asphalt binders for sustainable roadways. Advances in Human Aspects of Transportation.

[16] Airey G.D. (2004). Styrene butadiene styrene polymer modification of road bitumens. J. Mater. Sci..

[17] Airey G.D. (2003). Rheological properties of styrene butadiene styrene polymer modified road bitumens☆. Fuel.

[18] Xia T., Xu J., Huang T., He J., Zhang Y., Guo J., Li Y. (2016). Viscoelastic phase behavior in SBS modified bitumen studied by morphology evolution and viscoelasticity change. Constr. Build. Mater..

[19] Zhao K., Wang Q.Z., Zhuang H.Y., Li Z.Y., Chen G.X. (2022). A fully coupled flow deformation model for seismic site response analyses of liquefiable marine sediments. Ocean. Eng..

[20] Zhang F., Yu J. (2010). The research for high-performance SBR compound modified asphalt. Constr. Build. Mater..

[21] González O., Peña J.J., Muñoz M.E., Santamaría A., Pérez-Lepe A., Martínez-Boza F., Gallegos C. (2002). Rheological Techniques as a Tool To Analyze Polymer− Bitumen Interactions: Bitumen Modified with Polyethylene and Polyethylene-Based Blends. Energy Fuels.

[22] Wang S.J., Tai D.C. (2007). Evaluating indices for low-temperature performance of SBR modified asphalt binder. J. Chang. Univ..

[23] Kumar P., Mehndiratta H.C., Singh K.L. (2010). Comparative study of rheological behavior of modified binders for high-temperature areas. J. Mater. Civ. Eng. J. Mater. Civ. Eng..

[24] (2011). Standard Test Methods for Bitumen and Bituminous Mixture of Highway Engineering.

[25] (2004). Technical Specification for Construction of Highway Asphalt Pavements.

[26] Tian J., Guo L., Yin X., Wu W. (2018). The liquid-phase preparation of graphene by shear exfoliation with graphite oxide as a dispersant. Mater. Chem. Phys..

[27] (2018). StandardStandard Test Method for Separation of Asphalt into Four Fractions.

[28] Wang P., Dong Z., Tan Y., Liu Z. (2015). Investigating the interactions of the saturate, aromatic, resin, and asphaltene four fractions in asphalt binders by molecular simulations. Energy Fuels.

[29] Qu X., Liu Q., Guo M., Wang D., Oeser M. (2018). Study on the effect of aging on physical properties of asphalt binder from a microscale perspective. Constr. Build. Mater..

[30] Xu G., Wang H. (2017). Molecular dynamics study of oxidative aging effect on asphalt binder properties. Fuel.

[31] Yu X., Wang J., Si J., Mei J., Ding G., Li J. (2020). Research on compatibility mechanism of biobased cold-mixed epoxy asphalt binder. Constr. Build. Mater..

[32] Weiner P.K., Langridge R., Blaney J.M., Schaefer R., Kollman P.A. (1982). Electrostatic potential molecular surfaces. Proc. Natl. Acad. Sci. USA.

[33] Walubita L.F., Das G., Espinoza E., Oh J., Scullion T., Lee S.I., Garibay J.L., Nazarian S., Abdallah I. (2012). Texas Flexible Pavements and Overlays: Year 1 Report, Test Sections, Data Collection, Analyses, and Data Storage System.

[34] (2015). Standard Test Method for Viscosity Determination of Asphalt at Elevated Temperatures Using a Rotational Viscometer.

[35] (2015). Standard Test Method for Determining the Rheological Properties of Asphalt Binder Using a Dynamic Shear Rheometer.

[36] Asphalt Institute (1997). Superpave: Performance Graded Asphalt Binder Specification and Testing.

[37] (2010). Standard Method of Test for Determining the Rheological Properties of Asphalt Binder Using a Dynamic Shear Rheometer (DSR).

[38] Walubita L.F., Lee S.I., Faruk A.N.M., Scullion T., Nazarian S., Abdallah I. (2017). Texas Flexible Pavements and Overlays: Year 5 Report-Complete Data Documentation.

[39] Margenau H. (1939). Van der Waals forces. Rev. Mod. Phys..

[40] Dzyaloshinskii I.E., Lifshitz E.M., Pitaevskii L.P. (1961). The general theory of van der Waals forces. Adv. Phys..

